# Mass spectrometry-based proteome profiling of extracellular vesicles and their roles in cancer biology

**DOI:** 10.1038/s12276-019-0218-2

**Published:** 2019-03-15

**Authors:** Raju Bandu, Jae Won Oh, Kwang Pyo Kim

**Affiliations:** 1 Department of Applied Chemistry, Institute of Natural Science, Global Center for Pharmaceutical Ingredient Materials, Kyung Hee University, Yongin, Republic of Korea; 2 Department of Biomedical Science and Technology, Kyung Hee Medical Science Research Institute, Kyung Hee University, Seoul, Republic of Korea

**Keywords:** Cancer, Proteomics

## Abstract

Over the past three decades, extracellular vesicles (EVs) have arisen as important mediators of intercellular communication that are involved in the transmission of biological signals between cells to regulate various biological processes. EVs are largely responsible for intercellular communication through the delivery of bioactive molecules, such as proteins, messenger RNAs (mRNAs), microRNAs (miRNAs), DNAs, lipids, and metabolites. EVs released from cancer cells play a significant role in signal transduction between cancer cells and the surrounding cells, which contributes to the formation of tumors and metastasis in the tumor microenvironment. In addition, EVs released from cancer cells migrate to blood vessels and flow into various biological fluids, including blood and urine. EVs and EV-loaded functional cargoes, including proteins and miRNAs, found in these biological fluids are important biomarkers for cancer diagnosis. Therefore, EV proteomics greatly contributes to the understanding of carcinogenesis and tumor progression and is critical for the development of biomarkers for the early diagnosis of cancer. To explore the potential use of EVs as a gateway to understanding cancer biology and to develop cancer biomarkers, we discuss the mass spectrometric identification and characterization of EV proteins from different cancers. Information provided in this review may help in understanding recent progress regarding EV biology and the potential roles of EVs as new noninvasive biomarkers and therapeutic targets.

## Cancer: Protein-filled vesicles offer therapeutic and diagnostic targets

Tumor cells release tiny membrane-encapsulated packages known as extracellular vesicles containing proteins which could serve as prognostic disease biomarkers or therapeutic targets. Kwang Pyo Kim and colleagues from Kyung Hee University in Yongin, South Korea, review the use of mass spectrometry to profile the diversity of proteins found in these tumor-derived packages. The proteins found in these vesicles help mediate communication between cancer cells and their surrounding tissues. Different tumor types share many of these proteins in common, but there are differences in the protein profile related to cancer-associated biological processes such as metastasis and cell proliferation. Tests based on the proteins contained in these vesicles could help clinicians better identify, diagnose and treat specific cancers, although large, multicenter studies are needed to validate such strategies.

## Introduction

Extracellular vesicles (EVs) are membrane-surrounded vesicles released by numerous cell types into the extracellular microenvironment^1–3^. EVs are involved in cell–cell communication, coagulation, inflammation, immune response modulation, and disease progression^2,4–7^. Although EVs vary in size, biological function, and components, their significance in cancer progression and the potential use of EV molecules as novel cancer biomarkers has gradually increased. Cancer cells actively release EVs into neighboring tissues, and these EVs play dynamic roles in cancer progression and metastasis, invasion, angiogenesis, tumorigenesis, and immune modulation^8–10^. EVs released by cancer cells are usually chosen as a gateway in the search for biomarkers for a specific cancer type. Recent results pertaining to EV-cargo molecules, including proteins and miRNAs, are summarized in EVpedia (http://evpedia.info), an integrated and comprehensive database of EVs^11^.

The main focus of this review is proteome profiling of EVs using mass spectrometry (MS)-based proteomic approaches. We discuss the mass spectral characterization of isolated EV proteins from different cancers and the use of these proteins as predictive cancer biomarkers. Additionally, we summarize the key characteristics of enriched proteins in cancer-associated EVs as potential therapeutic targets and provide novel information on their roles in cancer development and progression. Information provided in this review may help in understanding recent progress regarding EV biology and the prospective roles of EVs as new noninvasive biomarkers and therapeutic targets, as well as emerging therapeutic opportunities and associated challenges.

## Classification of EVs

**Fig. 1:**
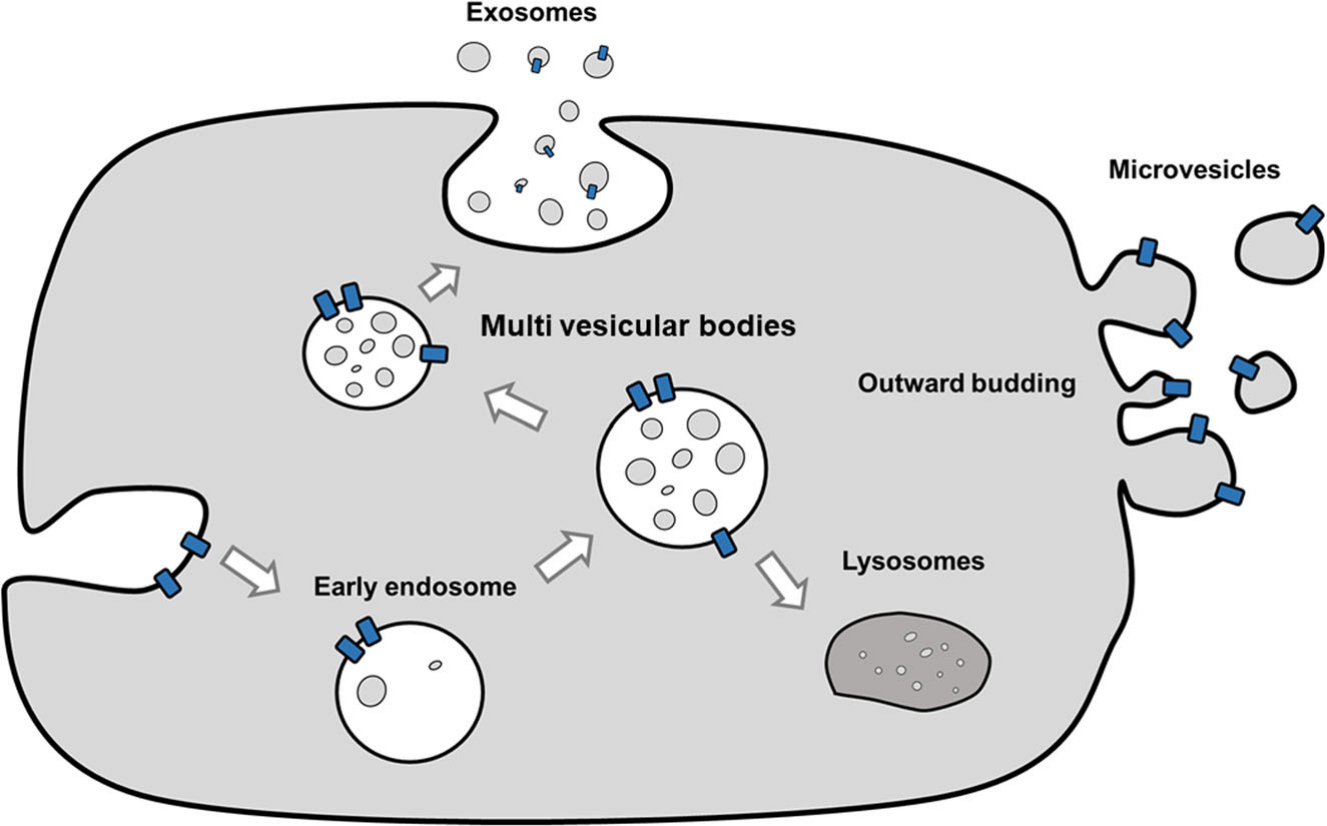
Biogenesis of four major subtypes of extracellular vesicles

EVs are small spherical vesicles that are secreted into the extracellular milieu by many cell types. The term “EV” was invented by the International Society of Extracellular Vesicles (ISEV) and is used to define all phospholipid bilayer-bound vesicles that are secreted by cells into the extracellular microenvironment, regardless of the differences in biogenesis, size, and composition^12,13^. The roles of EVs in different physiological and pathological processes have made them a novel field of research. EVs are categorized into several subtypes based on their size, density, shape, subcellular origin, function, and molecular cargo^14^. The four major subtypes of EVs are exosomes, microvesicles, apoptotic bodies, and oncosomes (Table 1 and Fig. 1). Exosomes are 30–200-nm-sized homogeneous membrane vesicles, and they form through the endosomal trafficking pathway^5,15,16^. Exosomes contain late endosomal markers, even though biochemically indistinguishable vesicles can bud directly from the plasma membrane^16,17^. They play critical roles in cell–cell communications, such as that occurring during the regulation of cell and tissue homeostasis, as well as in pathological conditions^18^. Microvesicles are 100–1000-nm-sized heterogeneous membrane vesicles that originate via outward budding and the fission of the plasma membrane due to dynamic interactions during phospholipid redistribution. Phospholipid distribution is controlled by aminophospholipid translocases^16,18–24^ and cytoskeletal protein contraction. Microvesicles are released mostly under cellular stress or in pathological processes^18^. Like exosomes, microvesicles transfer bioactive molecules into target cells. Apoptotic bodies (> 1 µm) are released by cells that undergo the apoptosis process or programmed cell death^18,24^, and they can be characterized by cellular organelles and DNA. Finally, the vesicles named “oncosomes” are much larger than most other EV types characterized to date (1–10 μm). Owing to their unusual size, large oncosomes might have unique properties in vivo and would provide novel opportunities for tumor profiling^25^.

**Table 1:** Brief classification of extracellular vesicles

EV subtype	Diameter	Biogenesis	Markers	References
Exosomes	30–200 nm	Released from multivesicular bodies within the endosomal network	Membrane transport and fusion proteins (annexins, GTPases, and flotillin), tetraspanins (CD9, CD63, CD81, and CD82), heat-shock proteins (Hsc70 and Hsp90), proteins involved in MVB biogenesis (Alix and Tsg101), lipid-related proteins and phospholipases, ESCRT, and MHC	^4, 5, 13– 15^
Microvesicles	100–1000 nm	Produced by direct budding from the cell membrane	Selectins, integrins (B1), metalloprotease surface phosphatidylserine, vesicle-associated membrane protein 3, CD34, CD40, CD45, glycophorin, or blood group antigens	^16– 22, 25, 58^
Apoptotic bodies	>1 µm	Released only by cells undergoing apoptosis or programmed cell death (apoptosis fragments)	Surface phosphatidylserinehistones, calnexin, cytochrome C, annexin V, C3b, and TSP	^4, 14– 22^
Oncosomes	1–10 µm	Non-apoptotic plasma membrane blebs shed by “ameboid” migrating tumor cells or from tumors	Cav-1, ARF6, Myr, Akt1, and HB‑EGF	^23^

EVs contain proteins, lipids, metabolites, and RNAs. However, the mechanisms by which these components enter EVs remain obscure. EVs are shed from almost all cell types and are present in biological fluids and conditioned cell culture media. EVs are involved in cell–cell communication, coagulation, inflammation, immune response modulation, and disease progression^4–7^. The functional roles of EVs in intercellular communication have made them of major interest in many scientific fields. The biomolecular composition of EVs could play a significant role in disease progression in several neurodegenerative diseases as well as in cancer.

## MS in EV proteome analyses

EV proteome analysis is a novel approach and is part of the growing interest in proteomics cancer research. Over the past three decades, many proteomics studies performed on EVs have elucidated their diverse roles. Large-scale proteomics datasets and protein-interaction networks have established significant relationships between EV proteins, which improves the understanding of vesicle biogenesis and pathophysiological roles^24,26,27^. Proteomic studies on EVs from different origins have also suggested a controlled protein-sorting mechanism and the random packaging of EV proteins from various cell types that contain common vesicular proteins. Furthermore, proteomic studies of EVs have produced a high-throughput vesicular proteome dataset from various cell types and body fluids^28^. Since EVs are normally isolated in small amounts, better sensitivity is required for their analysis. Liquid chromatography (nanoscale or ultra-high performance)–electrospray ionization tandem mass spectrometry (LC/ESI–MS/MS) is the most popular and versatile analytical technique to study the molecular contents of EVs. In particular, nano-ESI–MS/MS provides high sensitivity and resolution, allowing the detection, identification, characterization, and quantification of thousands of proteins from even a single EV sample. Similar to other biological fields, LC–MS/MS-based technological platforms have become the most popular fundamental tools for elucidating the structural and functional architecture of EVs. The fragment ions from ESI (positive- and negative-ion) tandem MS experiments provide the composition, unambiguous structural characterization, and proper identification of proteins present in various biological samples. Due to the high sensitivity and small initial sample volumes required for MS, MS-based proteomic analysis has increased the understanding of EV protein content. Several investigators^26,29–32^ have used ESI tandem MS experiments in combination with chromatographic methods (HPLC, UHPLC, UPLC, and nano LC) to profile and structurally characterize proteins in various cancer cells, tissues, biofluids, and biological samples, which have been summarized in Table 2.

**Table 2:** Summary of biomarker candidate proteins in extracellular vesicles from different cancers

Cancer type	Cancer-specific EV proteins	Isolation of EVs	Characterization	Sample source	References
Bladder cancer	EDIL-3	UC	WB, TEM	TCC, T24, SV-HUC, and urine	Beckham et al.^59^
		UC	TEM, NTA	TCCSUP, T24, UMUC3, RT3 SVHUC, and urine	Silvers et al.^60^
	ITGB1, ITGA6, CD36, CD44, CD73, CD10, MUC1, BSG, and 5T4	UC	NTA, TEM, and WB	HT1376, urine	Welton et al.^34^
		UC	NTA, TEM, and WB	T24, FL3, and SLT4	Jeppesen et al.^61^
Colon cancer	HGS	ExoQuick	NTA, TEM, and WB	HCT116	Sun et al.^62^
	Clstn1, VCP, and RuVB-like1 (O-GlcNAcylation)	UC	TEM, WB	CCD841, HT29, SW480, and SW620	Chaiyawat et al.^63^
	F2	BDG	NTA, TEM, and WB	SW480, SW620	Schillaci et al.^64^
	YWHAZ	UC		HCT116, patient colon tumor	Hillary et al.^65^
	ACACA	UC		Citrus-limon, SW480	Raimondo et al.^66^
		UC	NTA, WB	SW620	Guo et al.^67^
	EPCAM-CLDN7 and TNIK-RAP2A	BDG	NTA, TEM, and WB	SW480, SW620	Ji et al.^53^
		BDG	NTA, TEM, and WB	Patient tumor	Choi et al.^26^
		BDG	NTA, TEM, and WB	HT-29	Choi et al.^27^
	GPA33, CDH17, CEA, EpCAM, PCNA, EGFR, MUC13, MINK1, KRT18, MAPK4, CLDN (1, 3, and 7), CEP55, EFNB1, and EFNB2		TEM, WB	LIM1215 cells, urine, mast and cells	Suresh et al.^33^
		UC	NTA, TEM, and WB	Dks-8, DLD-1, and DKO-1	Demory et al.^68^
		BDG	NTA, TEM, and WB	SW480, SW620	Choi et al.^69^
	DKK4 and DNMT3A	UC	TEM, WB	SW480, SW480APC	Lim et al.^70^
	MAC2BP, ALIX, 14–3–3 isoforms, PFN1, CALU, and IL-8	UC	TEM	LIM1215 cells	Ji et al.^71^
Prostate cancer		UC	DLS, TEM	pc3-HSP27, HEK-293	Rauschenberger et al.^72^
		UC		PC3, DU145, VCaP, LNCaP, C4–2, and RWPE-1	Hosseini-Beheshti et al.^73^
	THBS1, GSN, and ITGB1	UC	WB	LNCaP	Soekmadji et al.^74^
	ITGB4 and VCL	UC, CD9 antibody magnetic beads	NTA, WB, and TEM	PC-3	Kawakami et al.^75^
	CD9	UC	TEM, WB	LNCaP, DUCaP PCa cells, and plasma	Soekmadji et al.^76^
		UC	TEM, WB, and NTA	DU145 Tax-Sen, DU145 Tax-Res	Kharaziha et al.^77^
	CD151 and CDCP1	UC		PC-3	Sandvig et al.^78^
	PDCD6IP, FASN, XPO1, and ENO1	UC, SG	TEM, WB	PNT2C2, RWPE1, PC346C, and VCaP	Duijvesz et al.^30^
		UC		Osteoblasts	Bilen et al.^79^
Lung cancer	AKT and ERK1/2	UC	WB	H3255, H1650	Van et al.^80^
	AKT1, GSK3B, EIF4E, MTOR, RELA, and RAS	BDG	TEM, WB	PC9, PC9R	Choi et al.^81^
	ALLIX, TSG101, CD3, EGFR, SRC, KRAS, and NRP1	PEG precipitation, UC, and BDG	TEM, WB	A54, HCC827, and HBEC	Clark et al.^39^
	P53 and EGFR	qEV	TRPS, TEM, and WB	30KTp53/EGFR	Lobb et al.^82^
HCC		ExoQuick	TEM, WB	Hep3B, 97 H, and LM3	Zhang et al.^83^
	RRAS, CD44, CDC42, and CLND3	UC	WB	HKCI-C3, HKCI-8, MHCC97L and MIHA	He et al.^84^
		UC	NTA, TEM, and WB	HepG2	Wang et al.^43^
		UC	WB, EM	Huh7.5.1 Huh7-ET	Ramakrishnaiah et al.^85^
Breast cancer		UC	TEM	Plasma, bone metastasis explant-conditioned media, and pleural effusion	Tucker et al.^86^
		vn96 affinity capture of EV	WB and TEM	SKBR3, MCF-7, and MCF-10a	Griffiths et al.^87^
		Free-flow electrophoresis		SKBR3 (hypoxia, normoxia)	Thomas et al.^88^
		UC	TEM, WB, and NTA	MCF-7, MDA-MB-231	Harris et al.^32^
	DEL-1			Plasma, MDA-MB-231	Moon et al.^89^
	EDIL3	BDG	NTA, TEM, and WB	MCF-7, MDA-MB-231	Lee et al.^29^
	IL-6, TNFa, GCSF, and CCL2	UC	TEM, WB, and FC	MCF10A, MDA-MB-231, and MCF7	Chow et al.^90^
	POSTN	UC, SG	NTA, TEM, and WB	MCF7, MDA-MB-231, 67NR, 4T1, and plasma	Vardaki et al.^91^
		UC	TEM, WB, and NTA	cal51 TNBC	Kavanagh et al.^92^
		UC	TEM, WB	MDA-MB-231 cells	Palazzolo et al.^37^
	MTDH and CP	UC	TEM, WB, and NTA	4T1, 4T1.2, 67NR, and 66cl4	Gangoda et al.^93^
		UC	TEM, DLS, and WB	VCaP	Domenyuk et al.^94^
Ovarian cancer		UC	TEM, WB	OVCAR3, OVCAR433, OVCAR5, and SKOV3	Sinha et al.^95^
		UC	WB, TEM	SKOV3, OVM	Escrevente et al.^96^
	G6PD and TKT	UC	WB, TEM	OVCA429, HO8910PM	Yi et al.^97^
		UC	TEM, WB	OVCAR-3, IGROV1	Liang et al.^41^
		UC	TEM, WB	SKOV3, CAOV3, and HUVEC	Yi et al.^98^
Pancreatic cancer	EGFR	UC	WB	BxPC3, MiaPaca2, and Panc1	Adamczyk et al.^99^
	ZIP4	SBI ExoQuick‐TC Kit	TEM, WB	PC‐1.0 (highly malignant), PC‐1 (moderately malignant)	Jin et al.^100^
	CEACAMs and ECM proteins	UC	TEM	Pancreatic duct fluid	Zheng et al.^101^
		UC, SG	EM	SOJ-6, BxPC-3, MiaPaCa-2, and Panc-1	Ristorcelli et al.^36^
	MYOF	UC	DLS, TEM, and WB	MDA-MB-231, MDA-MB-468, BT-549, Hs 578 T, MCF7, MCF-10A, ZR-75–1, BT-474, SK-BR-3, and CFPAC-1	Blomme et al.^102^
		BDG	TEM, WB	Panc1, BxPc3, MiaPaca2, and HPSC	Klein-Scory et al.^103^
	MIF	UC	NTA	PKCY, PAN02	Costa-Silva et al.^31^
		UC, SG	TEM, WB	Panc02, Panc02-H7 cells	Yu et al.^104^
	CLDN4, EPCAM, CD151, LGALS3BP, HIST2H2BE, and HIST2H2BF	UC	WB	13 human PDAC, 2 non-neoplastic cell lines	Castillo et al.^105^
	PLEC	UC, ExoQuick-TC	DLS, TEM, and WB	PDAC, C6 glioma cells, and HPDE	Shin et al.^106^
	WNT5B	BDG, SG	EM	CHO, Caco-2 cells	Harada et al.^46^
CCA		UC	TEM, WB	Human bile, H69 cell line	Chaiyadet et al.^107^
	S100A6, LUM, LCP1, YWHAZ, and VIM	UC	TEM	Hamster liver tissue, KKU055	Khoontawad et al.^108^
		UC	TEM, WB	KKU-100, KKU-M213, and H69	Dutta et al.^42^
Blood cancer	MARCKS	UC, SG	CM, FACS	K562, LAMA84	Taverna et al.^109^
	VCP	UC	TEM	U937, Mec1	Bosque et al.^56^
		UC	WB, FC	Primary CLL cells	Paggetti et al.^110^
	DNMT1 and HELLS	UC	NTA, FC	Molm-14, HL-60, and OP9 cells	Huan et al.^111^
	MHC-1, MHC-2, HSC70, HSP90, and ICMA-1	UC	TEM, WB	Raji cells	Yao et al.^40^
Oral cancer		ExoQuick	NTA, TEM	HUVEC, SCC15	Andrade et al.^112^
	NAP1	Ultrafiltration	NTA, TEM, WB, and CM	CAL 27, SCC-25	Wang et al.^44^
	HSP90	UC	TEM, NTA	HSC-3, HSC-3-M3	Ono et al.^113^

*WB* western blotting, *TEM* transmission electron microscopy, *NTA* nanoparticle tracking analysis, *DLS* dynamic light scattering, *TRPS* tunable resistive pulse sensing, *FC* flow cytometry, *CM* confocal microscopy

## EV proteomes in various cancers and biomarker discovery

Proteomic analysis of EVs has revealed significant changes in protein expression under various physiological and pathological conditions^26,29,30^. Characterization of these proteomic profiles may be useful in understanding disease pathogenesis and assisting in the discovery of new biomarkers for different diseases. The secretion of EVs from several types of tumor cells is a significant means of conditioning and altering the tumor microenvironment by malignant cells^31,32^. Multiple studies have reported that the secretion of EVs from cancer cells contributes to angiogenesis, metastasis, tumor formation, and disease progression^2,10,31,32^. EVs are more attractive sources of biomarkers because of their biological consequences and relatively noninvasive accessibility in a wide range of biological fluids. EVs have been studied in relation to numerous cancers, such as colorectal^27,33^, bladder^34^, prostate^35^, pancreatic^36^, breast^37^, gastric^38^, lung^39^, blood^40^, ovarian^41^, cholangiocarcinoma^42^, hepatocellular carcinoma^43^, and oral squamous cell carcinoma^44^ (Table 2), as well as cardiovascular diseases^45^ and malignancies of the central nervous system^21^. The proteomic analysis of EVs, specifically the analysis of their protein composition, may be helpful for further understanding the mechanisms of their biogenesis and their functional roles. Molecular communication between cancer cells and their stromal microenvironment is a key factor for cancer progression^46,47^. In conjunction with typical secretory pathways, it was proposed that these small membranous vesicles are alternate mediators of intercellular communication^19^. EVs carry an effector-rich proteome with the ability to control different functional properties of the recipient cell^48^. The protein composition of EVs from different sources was studied previously by using MS^30,49–54^, providing a robust basis for the identification of biomarker proteins in EVs for the purpose of quality control research. A thorough understanding of the protein composition of EV subtypes and the extent to which EV composition reflects the source cell composition is essential for further development of diagnostics and therapeutics. Although EVs are secreted by almost all cell types, some available data suggest the enhanced release of EVs under pathological conditions, such as cancer^55^. It is reasonable to expect that these vesicles may also play key roles in tumorigenesis since they can facilitate distant intercellular communication. Tumor-derived EVs typically carry tumor antigens, and functional proteins can be transferred to recipient cells through EVs^23,54,56^. A better understanding of the molecular bases underlying cancer invasion and metastasis is necessary to develop effective targets for therapy.

**Fig. 2:**
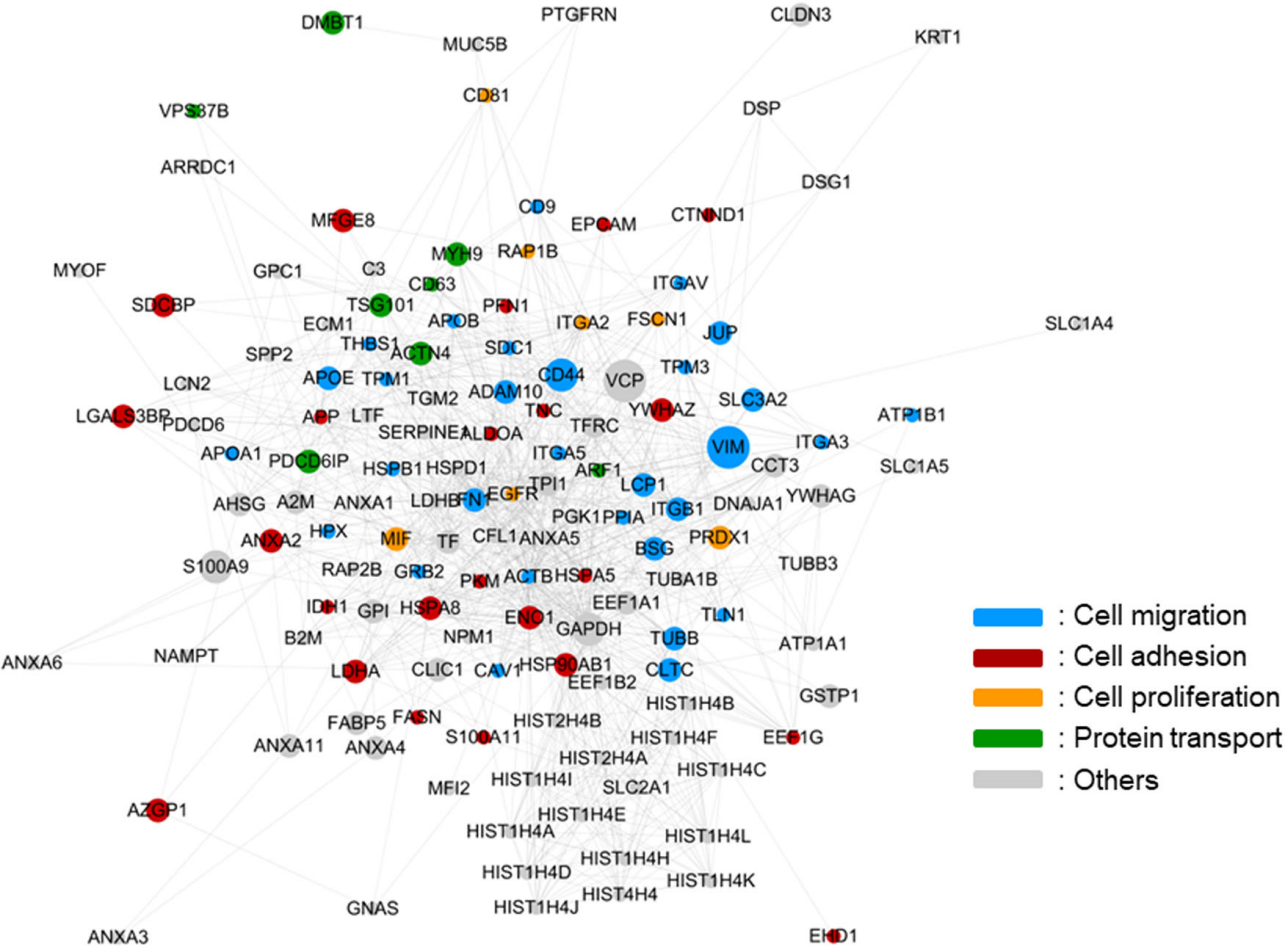
Protein–protein interaction network of differentially expressed extracellular vesicle proteins in cancer cell-derived EVs

EV proteins from many cancers have similar biological processes and functions. To understand the functions of differentially expressed proteins (DEPs) in cancer, we performed gene ontology analysis on a variety of DEPs^57^. As expected, the EV–DEPs from different cancer types were implicated in similar biological processes, such as cell adhesion, migration, and transport. Considering that EVs are potential metastasis factors, those proteins appear to be relevant for cancer metastasis or cancer cell proliferation. Of the 12 different cancers evaluated, we observed that DEPs that overlapped more than five times were primarily related to cancer metastasis or cancer cell proliferation, and many of the DEPs had strong interactions with each other (Fig. 2). Even though the selection of these DEPs from different cancers was biased, the roles of EVs in different cancers focused mainly on cell adhesion and cell migration.

## Conclusions

In this review, we summarized different EV studies to discuss the potential of EVs in cancer treatment. All studies discussed in this review indicated that the specific protein composition of various EVs has high potential for identifying different cancers. The majority of these studies revealed the relationship of cancer with changes in the protein contents of various body fluids. Moreover, we have highlighted the emerging roles of EVs in cancer, specifically their role in metastasis, which opens the possibility of the rapid translation of EV research for clinical applications in diagnosis, prognosis, and treatment. Ultimately, the majority of the investigations discussed in this review need further verification in large-cohort, multicenter clinical studies. In the future, highly reliable EV proteome data could be combined with well-developed current popular genomic and other “omics-” studies to provide extended knowledge of EVs from the perspective of systems biology approaches.

## Future perspectives

There are many perspectives on the potential contribution of EV research for the development of cancer therapeutics and diagnosis. EVs could play key roles in intercellular communication during cancer development, which may offer new therapeutic strategies for various cancers. EV protein composition in different body fluids reveals the overall condition of the patient and is also useful for screening the efficacy and toxicity of anticancer treatments. Additionally, EVs could be used as cancer vaccines and drug delivery components. Moreover, the inhibition of intercellular communication through EVs might provide opportunities to suppress tumor progression. In the near future, clinical applications of EVs could contribute to cancer management and treatment. However, before EV-targeted therapy can be applied in cancer, the identification of cancer-specific genes or molecules that are crucial for EV communication is necessary.
